# Psychological profile of Chinese peritoneal dialysis patients during the Omicron pandemic in 2022

**DOI:** 10.1186/s40359-024-01615-x

**Published:** 2024-03-01

**Authors:** Jin Qiu, Chunyan Zhang, Jingyuan Xie, Shan Lin, Hong Ren, Xiaomin Huang, Tian Xu

**Affiliations:** 1 Department of Nephrology, Ruijin Hospital, Shanghai Jiao Tong University School of Medicine, Shanghai, China; 2 Department of Nephrology, Shanghai TCM-Integrated Hospital, Shanghai University of Chinese Medicine, Shanghai, China

**Keywords:** Omicron, Peritoneal dialysis, Psychological status

## Abstract

**Objective:**

The aim of this study was to determine the psychological status of peritoneal dialysis (PD) patients who were blocked during the 2022 Omic Pandemic in Shanghai.

**Methods:**

This was an observational and cross-sectional study. We selected 172 PD patients from the peritoneal dialysis center of Ruijin Hospital, Shanghai Jiao Tong University School of Medicine, during the quarantine of the Omicron pandemic in Shanghai from April to May 2022. General data and biochemical indices were collected. The Kidney Disease Quality of Life (SF-36) questionnaire was used to evaluate the psychological state of the patients during the quarantine.

**Results:**

According to the assessment of the SF-36 scale, the physiological and psychological health status of PD patients was better than that before quarantine (*P* < 0.05). According to the comparison of biochemical indices, the high-density lipoprotein, total cholesterol and body mass index (BMI) levels were lower in patients after quarantine than before quarantine, while the blood phosphorus, blood calcium and haemoglobin levels were greater after quarantine (*P* < 0.05). Logistic regression analysis revealed that health changes were positively correlated with age of penetration (years) (OR = 1.031, 95% CI = 1.005–1.058); however, physiological function was negatively correlated with sex (OR = 0.198, 95% CI = 0.044–0.899). Energy was significantly positively correlated with closed-loop time (OR = 1.063, 95% CI = 1.001–1.128) (*P* < 0.05). There were no significant differences in biochemical indices or quality of life between APD patients and non-APD patients (*P* > 0.05). According to the results of the abstract independent sample T test, when comparing the various dimensions of the SF-36 scale, for the dimensions of physiological function, pain and energy, the PD patients were better than the HD patients were (*P* < 0.05). Similarly, for the dimension of physiological function, the HD patients were better than the PD patients were (*P* < 0.05). During the quarantine period from April to May in Shanghai, the infection rate of PD patients was lower than usual (*P* < 0.05).

**Conclusions:**

During the Omicron pandemic in Shanghai in 2022, PD patients exhibited relatively stable psychological and physiological states and a low infection rate. Compared with HD patients, PD patients had better adaptability. Especially in the context of the COVID-19 pandemic, peritoneal dialysis has more advantages.

## Introduction

Chronic kidney disease (CKD) is a global health problem. Epidemiological investigations have shown that the prevalence of CKD worldwide is approximately 14.3% [1] and that in China, it is approximately 10.8% [2]. After CKD progresses to end-stage renal disease (ESRD), patients depend on dialysis or kidney transplantation to maintain life. Mental illness is a common complication in ESRD patients. When patients receive dialysis treatment, the incidence of mental illness greatly increases, and these mental health problems directly affect their dialysis quality. In addition to the existing factors of their own disease, changes in the social environment also have a significant impact on the mental health of patients [3]. On March 11, 2020, the World Health Organization announced the novel coronavirus (severe acute respiratory syndrome coronavirus 2) pandemic infection (COVID-19). Since the outbreak of the novel coronavirus pneumonia, this sudden public health event has had an enormous impact on people’s quality of life and mental health [4, 5]. Recent studies have shown that, especially for patients with chronic diseases, during the COVID-19 outbreak, the prevalence of depressive symptoms among quarantined individuals and medical staff in China was 6.21% and 6.46–50.7%, respectively [6]. However, to date, no detailed studies have been conducted on the mental health of peritoneal dialysis (PD) patients facing an epidemic. Especially in the first half of 2022, during the epidemic period of the COVID-19 Omicron mutant in Shanghai, Shanghai implemented comprehensive quarantine management from April to May 2022. In view of this background, we focused on the psychological characteristics of peritoneal dialysis patients during this event in this study to better understand the impact of social and psychological factors on the mental health of this population.

## Materials and methods

### Research subjects

We selected peritoneal dialysis patients at the Peritoneal Dialysis Center of Ruijin Hospital, Shanghai Jiao Tong University School of Medicine, during the quarantine of the Omicron Pandemic in Shanghai from April to May 2022.

### Research criteria

#### Inclusion criteria

1. All patients met the diagnostic criteria for related diseases and received peritoneal dialysis;
2. Agreeing to participate in the investigation;
3. Patients who received peritoneal dialysis for more than 3 months;
4. Have normal communication ability;
5. Patients were able to read or understand the content of the questionnaire and be able to communicate orally or in writing.

#### Exclusion criteria

1. Patients with complications that may affect the accuracy of the scale assessment, such as patients with severe heart, liver, brain or other diseases;
2. Patients with mental illness or cognitive dysfunction;
3. severe gastrointestinal disease;
4. Follow-up the patients who died;
5. Peritonitis occurred half a year before enrollment.

### Research methods

We collected data and various laboratory indices before and after the epidemic within one month. During the investigation, Shanghai developed and implemented strict prevention and control measures. The short form-36 health survey (SF-36) [7] is the most widely used quality of life measurement tool in the world. The NRS-2002 is specifically used to evaluate the quality of life of dialysis patients. These scores can be divided into mental scores (MCSs) and physiological scores (PCSs). The questionnaire was administered by trained physicians face-to-face, and the relevant items were explained to the patients. We used the SF-36 quality of life questionnaire and the home situation questionnaire during the epidemic period, and a total of 172 PD patients completed the relevant questionnaires. The form was completed by the patient himself, under the guidance of a dedicated researcher, or assisted by the researcher if the patient could not complete the form independently due to illness or cultural reasons.

In addition, we collected general information from the patients, including age, sex, marital status, education level, dialysis age, complications, drugs, etc. This study was approved by the Ethics Committee of Ruijin Hospital (2015) Ethical Approval No. 105, and all patients or their families signed informed consent forms. A history of cardiovascular disease (CVD), diabetes, hypertension, hyperuricemia, hyperlipidemia and anemia was recorded in detail.

### Statistical analyses

All the data analyses were performed using SPSS 25.0 statistical software. Differences were deemed to be significant at *p* < 0.05. Categorical variables are expressed as the frequency (n) and percentage (%). Normally distributed quantitative variables are expressed as the mean ± standard deviation, or nonnormally distributed quantitative variables are expressed as the median (25th, 75th). Categorical variables were tested by the chi-square test. Normally distributed variables were compared using a t test. Nonnormally distributed variables were compared using the Kruskal‒Wallis H test. For the independent sample t test without original data, an abstract independent sample t test was adopted. Logistic regression analysis was used to determine the factors influencing quality of life, and odds ratios (ORs) and relative 95% CIs were estimated.

## Results

### Comparison of patients’ general conditions

A comparison of the patients’ general conditions, including age, sex, education level, marital status, dialysis age and primary renal disease, is shown in Table 1. A total of 172 patients were included in this study; 92 were male, and 80 were female, with an average age of 53.33 ± 14.82 years.

**Table 1:** Social and demographic characteristics of the participants (*n* = 172)

Demographic data		
Age (years)	53.33 ± 14.82	
Sex		
Male	92(53.49%)	
Female	80(46.51%)	
Education level		
Junior High School and below	45(26.16%)	
Senior high school	49(28.49%)	
College and undergraduate	71(41.28%)	
Postgraduate or above	7(4.07%)	
Marital status		
Married	143(83.14%)	
Unmarried	19(11.05%)	
Divorced	6(3.49%)	
Widowed	4(2.32%)	
Dialysis age		
≦2 years	84(48.84%)	
More than 2 years	88(51.16%)	
Primary renal disease		
Diabetes nephropathy	27(15.70%)	
Hypertensive nephropathy	13(7.56%)	
Chronic glomerulonephritis	118(68.60%)	
Other types	14(8.14%)	

### Evaluation of the SF-36 scale score in PD patients before and after lockdown

A total of 172 questionnaires were distributed, and 172 effective questionnaires were recovered, for an effective recovery rate of 100%. Table 2 shows the assessment of each dimension of the SF-36 scale in PD patients during quarantine. As shown in Table 2, the general health status, social function, health changes, body pain and energy of patients after quarantine were significantly better than those before quarantine (*P* < 0.05). The physiological and psychological scores of the PD patients were significantly better than those before quarantine (*P* < 0.05).

**Table 2:** Evaluation of the SF-36 score in patients before and after the lockdown

	Before the lockdown(*n* = 172)	After the lockdown (*n* = 172)	*P* value
General Health(GH)	38.84 ± 18.73	44.73 ± 19.15	0.005
Social Functioning(SF)	55.17 ± 22.97	65.32 ± 26.77	0.0001
Role-Physical(RP)	0(0, 50)	25(0, 75)	0.28
Reported HealthTransition (HT)	45.31 ± 30.75	54.69 ± 25.69	0.003
Physical Functioning(PF)	66.85 ± 25.86	70.74 ± 22.10	0.162
Bodily Pain(BP)	62.82 ± 27.91	76.20 ± 23.99	0.0001
Vitality(VT)	51.76 ± 21.37	61.48 ± 18.69	0.0001
Role-Emotional(RE)	33(0, 100)	50(0, 100)	0.195
Mental Health(MH)	68.50 ± 19.86	64.75 ± 17.73	0.068
Physiological Score(PCS)	49.55 ± 18.65	56.65 ± 18.32	0.0001
Mental Score(MCS)	56.02 ± 21.59	60.84 ± 20.62	0.024

### Comparison of biochemical indices of PD patients before and after quarantine

We collected relevant biochemical indices of PD patients before and after quarantine, including routine blood, renal function, electrolytes, iron metabolism, blood glucose, blood lipids, immunoreactive parathyroid hormone (iPTH), and inflammatory indicators. As shown in Table 3, the serum levels of HS-cTn, IL-6, BUN, HDL-C, total cholesterol (TC), nPCR and BMI were significantly lower after quarantine than before quarantine (*P* < 0.05). P, Ca, Hb, Glu, HCT, and 25-OH-VD increased significantly after quarantine compared with before quarantine (*P* < 0.05).

**Table 3:** Changes in biochemical indices of PD patients before and after quarantine

Biomarker	Before quarantine (*n* = 172)	After quarantine (*n* = 172)	*P* value
HS-cTn, ng/ml	11.9(6.5, 21.6)	10.6(5.7, 18.1)	0.042
P, mmol/l	1.60 ± 0.39	1.69 ± 0.42	0.021
Ca, mmol/l	2.30 ± 0.16	2.33 ± 0.17	0.03
Hb, g/l	111.89 ± 13.78	116.40 ± 15.14	0.003
BUN, mmol/l	19.97 ± 5.47	18.31 ± 4.96	0.0001
Glu, mmol/l	5.58 ± 1.53	6.00 ± 3.02	0.002
HCT, %	33.38 ± 4.33	34.31 ± 4.65	0.042
HDL-C, mmol/l	1.15 ± 0.39	1.08 ± 0.34	0.0001
TC, mmol/l	4.43(3.78, 5.42)	4.34(3.81, 5.11)	0.002
25- OH-VD, ng/ml	19.56(15.09, 25.49)	23.11(18.17, 29.98)	0.0001
nPCR	0.96 ± 0.19	0.91 ± 0.20	0.001
BMI, kg/m^2^	23.08 ± 3.52	22.77 ± 3.48	0.0001

Abbreviations: HS-cTn, high sensitivity troponin; IL-6, interleukin 6; BUN, blood urea nitrogen; HDL-C, high density lipoprotein; TC, total cholesterol; BMI, body mass index; P, serum phosphorus; Ca, serum calcium; Hb, hemoglobin; Glu, blood glucose

### Logistic analysis of the SF-36 scale dimensions in PD patients

**Fig. 1:**
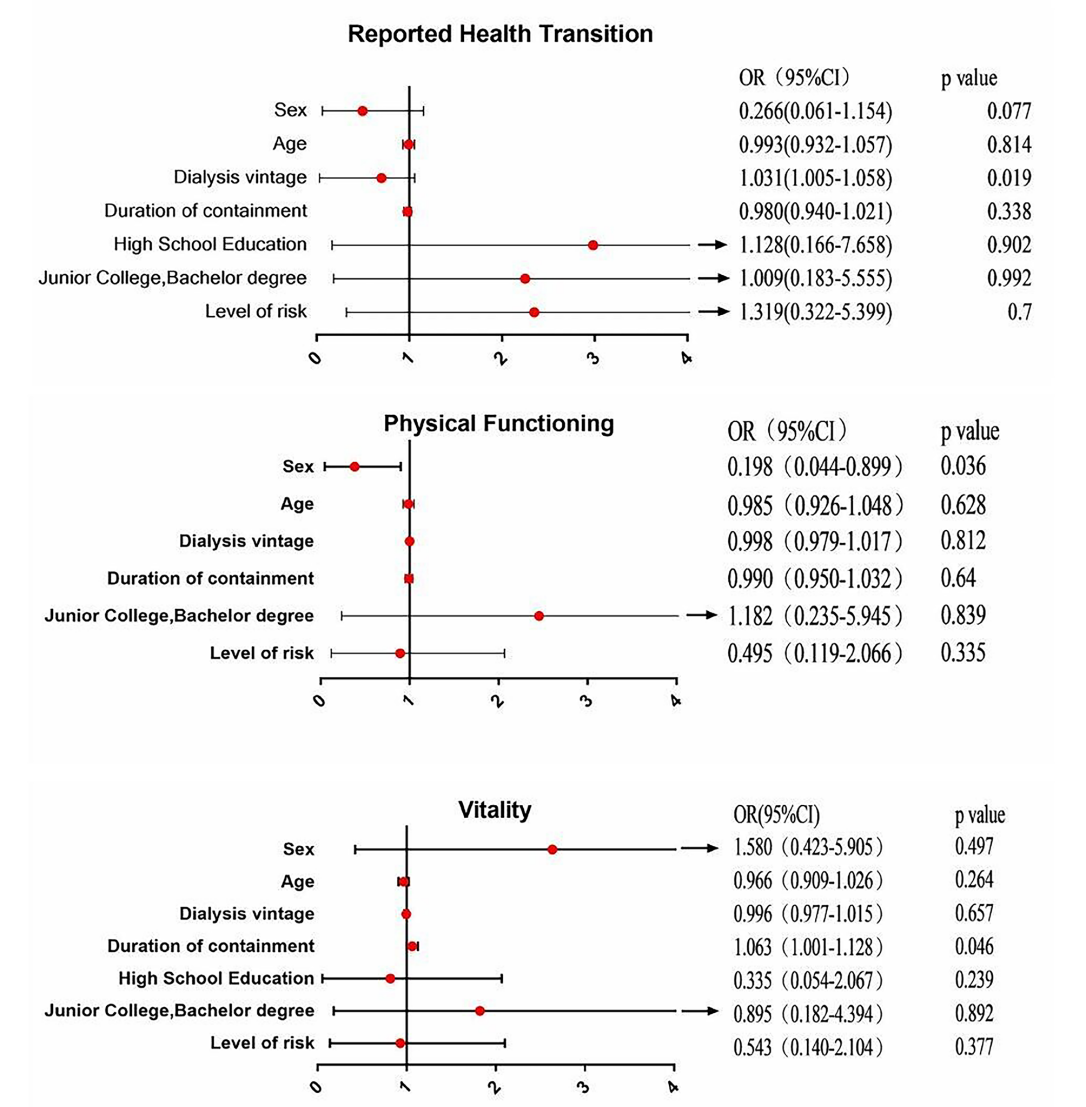
Logistic analysis of the SF-36 scale scores Note: Fig. 1 shows the logistic analysis of health changes (HT), physiological function (PF) and energy (VT) on the SF-36 scale in PD patients

Factors such as age, sex, dialysis vintage, duration of quarantine, education level, marital status and risk level were considered independent variables for logistic regression analysis on the impact of patients’ quality of life. The results showed that health changes (HT), physiological function (PF) and energy (VT) were significantly related to these changes. As shown in Fig. 1, health changes were positively correlated with age at dialysis (OR = 1.031, 95% CI = 1.005–1.058; *P* = 0.019); physiological function was negatively correlated with sex (OR = 0.198, 95% CI = 0.044–0.899; *P* = 0.036); and energy was positively correlated with closed-loop time (OR = 1.063, 95% CI = 1.001–1.128; *P* = 0.046).

### Comparison of the SF-36 score between PD patients and HD patients

**Fig. 2:**
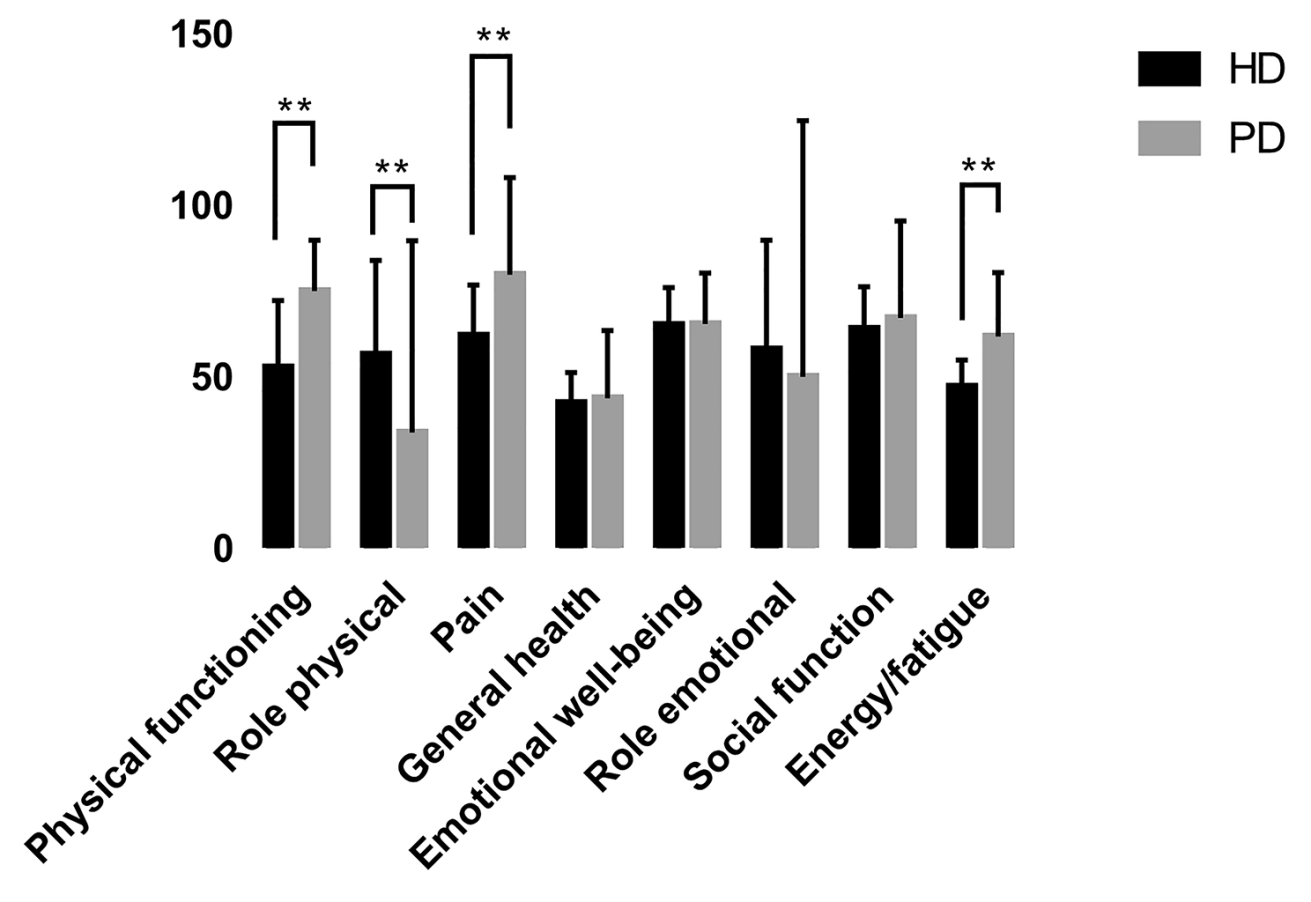
Comparison of SF-36 scores between PD patients and HD patients. Note: ** represents *p* < 0.05

Figure 2 shows the comparison of the SF-36 scores between PD patients and HD patients. With reference to the SF-36 scores of HD patients in our hospital [8], we compared the SF-36 scores through the use of an independent sample t test [9–11]. As shown in Fig. 2, in the PF (physiological function), BP (pain), and VT (energy) dimensions, the PD patients were significantly superior to the HD patients (*P* < 0.05). In the RP (physiological function) dimension, HD patients were significantly superior to PD patients (*P* < 0.05).

### Evaluation of the infection rate in PD patients during quarantine

According to the requirements of the 2022 edition of the International Society of Peritoneal Dialysis (ISPD) guidelines [12], the infection rate of peritoneal dialysis patients should be lower than 0.4 per patient-year. Table 4 shows the evaluation of the peritoneal dialysis infection rate during quarantine. As shown in Table 4, the peritoneal dialysis infection rate of patients during quarantine from April to May in Shanghai was 0.0025 per patient-month. In combination with the previous incidence of peritonitis in the peritoneal dialysis center of Ruijin Hospital, which was 0.13 times per patient-year, the incidence of peritonitis during the epidemic was significantly lower than usual (*P* < 0.05).

**Table 4:** The infection rate of peritoneal dialysis during quarantine

	n	preepidemic	during the epidemic	χ^2^	*P*
Peritonitis (case)	400	9	2	4.517	0.034
Infection rate (times/patient month)		0.0108	0.0025		

## Discussion

With the transformation of the health concept from a biological health model to a biopsychosocial health model, the treatment of end-stage renal disease is no longer limited to life maintenance and symptom relief but rather aimed at improving and restoring patients’ physical, psychological and social activities in an all-round way. Therefore, quality of life has gradually become an important index for comprehensive evaluation of dialysis treatment. The rapid spread of the COVID-19 pandemic around the world has had an enormous impact not only on healthcare systems but also on individuals. The latest research shows that patients with end-stage renal disease have a significantly increased risk of morbidity and death due to COVID-19, with a mortality rate as high as 20-30% [13–17]. The mental health pressure of patients receiving dialysis treatment is increased [18, 19]. Several studies believe that the high incidence of mental health problems in HD patients is related to poor quality of life and that feelings of happiness are negatively affected. According to relevant reports at home and abroad, the current psychological health assessment of dialysis patients is focused mainly on HD patients, while the psychological health assessment of PD patients has been limited during epidemics. Therefore, we carried out a study on the psychological health of PD patients facing the epidemic.

Research by McKeaveney Clare et al. [20] showed that HD patients in Northern Ireland reported a worse sense of well-being and an increased incidence of anxiety and depression during the pandemic compared to those before the pandemic. A recent study in the Netherlands [21] investigated the mental health status of dialysis patients during the COVID-19 epidemic. Compared with that in pre-COVID-19 patients, the mental health of dialysis patients was not affected by the pandemic. However, a study from Thailand reported [22] that PD patients had a moderate to good mental state during the lockdown period, and the analysis suggested that religion and personal beliefs were important influencing factors.

To our surprise, the results of our study showed that the physiological and psychological states of PD patients were better than those before quarantine. Through further communication with patients, we learned that during quarantine, PD patients received more company and care at home, while in the past, their family members were usually busy at work and neglected to communicate. After being accompanied by family members, the patients’ mood became more stable, they felt more secure, and their psychological and physical status improved.

On the other hand, the relevant studies of Sousa Helena et al. showed that [23] compared with those before quarantine, the blood phosphorus level of dialysis patients was significantly greater, and the serum ALB level was significantly lower. Because of the COVID-19 pandemic and relevant restrictive measures, it was difficult for patients to maintain their diet during the closure period. Due to the lack of corresponding materials, the intake of dietary nutrients decreased, and the intake of phosphorus-containing foods (such as bread and milk) increased, leading to an increase in blood phosphorus and a decrease in serum ALB.

Like the results of the present study, our study showed that the blood phosphorus level was also greater in patients after quarantine than before quarantine. In addition, the blood calcium and haemoglobin levels were greater than those before quarantine, and the high-density lipoprotein (HDL) level, total cholesterol level and BMI were lower than those before quarantine. We also considered that during quarantine, most of the food distributed to residents by the government was canned, frozen or other relevant foods that were easy to store, and the increased intake of these foods led to increased blood phosphorus. In addition, inadequate nutrient intake led to decreased HDL, total cholesterol, and BMI. Because of the lockdown, patients could not go to the hospital outpatient clinic for regular follow-up or adjustment of medication, while most patients with CKD had renal anaemia and calcium and phosphorus metabolism disorders [24–25]. During the period of confinement, the patients were continuously taking drugs to correct anaemia and regulate calcium and phosphorus metabolism, so haemoglobin and blood calcium were increased.

Javed S et al. suggested [26] that, compared with that before the epidemic, the level of anxiety associated with death increased in HD patients. During the closure period, HD patients generally had moderate death anxiety, and approximately one-third (31.6%) of HD patients experienced death anxiety from high to very high levels. Through logistic regression analysis, Williamson EJ et al. suggested that [27] the demographic and clinical risk factors leading to greater psychopathology symptoms in HD patients were female sex, older age (> 75 years old), married status, lower educational status, living with family, and the need for self-care and social support. Factors such as increased fear of death, decreased quality of life and life expectancy, increased psychological stress, and feelings of helplessness and hopelessness among dialysis patients due to COVID-19 increase the risk of suicide; in particular, HD patients older than 75 years who need self-care and social support are at increased risk of psychological distress, death anxiety and suicide [28].

In contrast to the risk factors for HD patients, the logistic regression analysis in our study showed that the factors related to the psychological and physiological states of PD patients were female sex, age at dialysis and length of time in the closed loop. Quarantine had a shorter duration of penetration, while quarantine had a greater impact on patients with longer closed-loop times. PD patients had autonomy in dialysis management. Family dialysis minimized exposure to COVID-19 infection, and participants maintained their health through family support, appropriate activities and exercise. In addition, contrary to the findings of previous studies, women’s psychological state is better than that of men, which may be related to the fact that most Chinese women are diligent in housework at home. Xiong et al. [29] showed that moderate exercise can improve the overall health status of dialysis patients, as indicated by physical symptoms and functions; is also conducive to mental health; and can alleviate depression and anxiety and improve the mood of dialysis patients [30, 31]. It is beneficial for improving cardiovascular function, blood pressure, nutritional status and dialysis quality and can effectively reduce fatigue and ameliorate sleep disorders [32].

Paraskevi et al. [33] showed that, through multidimensional investigation, HD patients had poor quality of life in many aspects of the environment and social relationships before the epidemic, and HD patients had more suicidal tendencies and sleep problems than PD patients did. Russo Gaspare Elios [34] et al. showed that, compared with HD patients, PD patients have better physical and mental health conditions, especially for elderly patients; moreover, PD patients have a better quality of life, while HD patients are more likely to suffer from depression.

Especially during the epidemic quarantine period, the psychological difference between PD patients and HD patients was more obvious. The results of our study showed that, in terms of psychological state, PD patients were generally better than HD patients were, which may be related to the greater dependence of HD patients on medical institutions, while PD patients themselves play an important role in dialysis. HD patients must attend dialysis centers three times a week for treatment, and if a patient in the dialysis center is diagnosed with COVID-19, all members of the dialysis center may have to be quarantined. In contrast, HD patients have greater psychological stress. In addition, in terms of preventive measures, PD patients pay more attention to hygiene, better hand washing and mask wearing compliance than HD patients do, possibly because PD patients are taught to always wear masks and maintain hand hygiene when exchanging peritoneal dialysis fluid [35]. Therefore, these patients have developed such habits and can take preventive measures, which may alleviate their psychological distress.

Peritonitis associated with peritoneal dialysis is one of the most common complications in PD patients and is also the main cause of technical failure in PD patients. According to reports from foreign peritoneal dialysis centres, the incidence of peritonitis is 0.06 ∼ 1.66 times per patient-year, and that of large peritoneal dialysis centres in China is 0.14 ∼ 0.17 times per patient-year [36]. The incidence rate of peritonitis in the peritoneal dialysis center of Ruijin Hospital is 0.13 times per patient-year. The results of our study suggest that the incidence rate of peritonitis during the epidemic was lower than usual, which may be related to the fact that PD patients pay more attention to personal hygiene and disinfection during family dialysis because they are afraid of going back and forth to the hospital.

### Summary

In summary, the above research results show that peritoneal dialysis has the advantages of requiring home treatment, avoiding cross-infection, ensuring autonomy and simplicity, etc. During the novel coronavirus epidemic, PD patients exhibit relatively stable psychological and physiological states and a low infection rate. Compared with HD patients, PD patients have better adaptability and emotional states. Especially in the context of the COVID-19 pandemic, peritoneal dialysis has more advantages. However, there are still some shortcomings in this study, which analysed only a single scale. It may be more effective to evaluate and analyse the psychological state of PD patients at multiple levels by analysing multiple scales.

## Data Availability

The datasets generated and analysed during the current study are not publicly available due patient privacy issues but are available from the corresponding author on reasonable request.
